# A pilot exploration of multi-omics research of gut microbiome in major depressive disorders

**DOI:** 10.1038/s41398-021-01769-x

**Published:** 2022-01-10

**Authors:** Haoyang Zhao, Kangyu Jin, Chaonan Jiang, Fen Pan, Jing Wu, Honglin Luan, Zhiyong Zhao, Jingkai Chen, Tingting Mou, Zheng Wang, Jing Lu, Shaojia Lu, Shaohua Hu, Yi Xu, Manli Huang

**Affiliations:** 1 Department of Psychiatry, the First Affiliated Hospital, Zhejiang University School of Medicine, Hangzhou, 310003 China; 2 The Key Laboratory of Mental Disorder Management in Zhejiang Province, Hangzhou, 310003 China; 3 Brain Research Institute of Zhejiang University, Hangzhou, 31003 China; 4 Zhejiang Engineering Center for Mathematical Mental Health, Hangzhou, 310003 China; 5 The M.O.E. Key Laboratory of Laboratory Medical Diagnostics the College of Laboratory Medicine Chongqing Medical University, Chongqing, 400016 China; 6 Department of Psychiatry, Wen Zhou seventh People’s Hospital, Wenzhou, 325006 China; 7 Key Laboratory for Biomedical Engineering of Ministry of Education, Department of Biomedical Engineering, College of Biomedical Engineering & Instrument Science, Zhejiang University, Hangzhou, 310027 Zhejiang Province China

**Keywords:** Depression, Diagnostic markers

## Abstract

The pathophysiology of major depressive disorder (MDD) remains obscure. Recently, the microbiota-gut-brain (MGB) axis’s role in MDD has an increasing attention. However, the specific mechanism of the multi-level effects of gut microbiota on host metabolism, immunity, and brain structure is unclear. Multi-omics approaches based on the analysis of different body fluids and tissues using a variety of analytical platforms have the potential to provide a deeper understanding of MGB axis disorders. Therefore, the data of metagenomics, metabolomic, inflammatory factors, and MRI scanning are collected from the two groups including 24 drug-naïve MDD patients and 26 healthy controls (HCs). Then, the correlation analysis is performed in all omics. The results confirmed that there are many markedly altered differences, such as elevated Actinobacteria abundance, plasma IL-1β concentration, lipid, vitamin, and carbohydrate metabolism disorder, and diminished grey matter volume (GMV) of inferior frontal gyrus (IFG) in the MDD patients. Notably, three kinds of discriminative bacteria, Ruminococcus bromii, Lactococcus chungangensis, and Streptococcus gallolyticus have an extensive correlation with metabolome, immunology, GMV, and clinical symptoms. All three microbiota are closely related to IL-1β and lipids (as an example, phosphoethanolamine (PEA)). Besides, Lactococcus chungangensis is negatively related to the GMV of left IFG. Overall, this study demonstrate that the effects of gut microbiome exert in MDD is multifactorial.

## Background

Major depressive disorder (MDD) has a high incidence and it’s a severe mental disorder causing suicide attempts [1]. MDD is ranked as the single largest contributor to health loss by WHO [2]. Though the mechanism of MDD remains unclarified, it is believed to be a heterogeneous etiology. The current theories have shown that genetics, neuro-endocrinology, neuro-immunity, structural and functional abnormalities of brain regions all play an important role in the pathophysiology of MDD, but the mechanism of MDD is poorly defined. Thus, it’s urgent to more thoroughly understand the pathogenic mechanism of MDD.

Recently, more and more attention has been paid to the gut microbiome in the pathogenesis of neuropsychiatric diseases [3–5]. Growing evidence has shown a strong association between MDD and microbiota-gut-brain (MGB) axis dysfunction [6–9]. Studies have shown that relative to healthy individuals, MDD patients showed reduced Bifidobacterium [5, 10], Lactobacillus [10], Firmicutes [11, 12], and Lachnospiraceae [7] ‘’and increased, Actinobacteria [11–13], Bacteroidetes [7, 11–14], and Proteobacteria [12]. Rats or mice that underwent fecal microbiota transplantation from MDD patients have shown depression‐like behaviors, which provided further insight into the role of the MGB axis in depression [3, 15–17]. The MGB axis exerts its effects through immune system activation (e.g., inflammatory cytokines and chemokines), neurotransmitters production (e.g., serotonin, gamma-aminobutyric acid [GABA] and glutamate), and its metabolites (short-chain fatty acids (SCFA) and key dietary amino acids, such as tryptophan (TRP)) [18]. In addition, the enteric nervous hypothesis explains that the gut microbiome through the autonomic nervous system, especially the vagus nerve achieves biphasic communication between the gut and the CNS [15, 19].

Some studies suggested that altered gut microbes interact with changed brain structure. In schizophrenia patients, the regional homogeneity (ReHo) indexes in the right superior temporal cortex, the left cuneus, and the right middle temporal cortex were negatively correlated with the abundance of the genus Roseburia [20]. Several studies have explored the neuroendocrine hypothesis of the MGB axis. An animal study revealed that significant correlations between amino acids, including L-threonine, isoleucine, alanine, serine, tyrosine, and oxidized proline and the altered fecal microbiota, mainly contain genera Prevotella, Oligella, Blautia, Phascolarctobacterium, Faecalibacterium, and Desulfovibrio [21]. Additionally, increased pro‐inflammatory signaling enhanced the number of pro-inflammatory bacteria (e.g., Proteobacteria, Allistipes, Prevotella, Oscillibacter, Actinobacteria) and decreased the anti‐inflammatory bacteria (e.g., Firmicutes, Faecalibacterium, Lachnospiraceae, Bacteroidetes) in MDD [22]. This finding suggests a potential link between immunology and gut microbiota.

However, several and not yet fully understood mechanisms are involved in this complex bidirectional network in health and during diseases, especially in major depressive disorder (MDD). The specific mechanism of the multi-level effects of gut microbiota on host metabolism, immunity, and brain structure is unclear. Multi-omics approaches based on the analysis of different body fluids and tissues using a variety of analytical platforms have the potential to provide a deeper understanding of MGB axis disorders [18]. Little previous research has focused on species level [14]. Besides, the search for multi-omics integration is the trend of diseases with heterogeneous etiology, especially neuropsychiatric diseases [15, 23, 24].

Therefore, this study explores the connection between the microbiome metagenomics, immunology, metabolomics, and brain structure in MDD. We recruited drug-naïve MDD patients and HCs, and collected their data of metagenomics, metabolomic, inflammatory factors, and MRI scanning. Subsequently, correlation analysis was performed to explore the relationship with each omics in MDD.

## Methods

### Participants

Patients were recruited from the First Affiliated Hospital of Zhejiang University between 2019 and 2020. The inclusion criteria for patients to enter this study were as follows: (1) age between 18 to 45 years; (2) meeting the criteria of the Diagnostic and Statistical Manual of Mental Disorder­5 (DSM-5) for treatment-naïve patients with current unipolar MDD; (3) score of the 17­item Hamilton Depression scale (HAMD-17) is greater than 17 points; (4) of Han ethnicity and right­handedness; (5) junior high school education or above; (6) voluntary in this study, signed written informed consent. The exclusion criteria included: (1) the patients with the treatment in any form prior to the study; (2) any other current or past psychiatric disorders, except MDD; (3) any secondary mental disorders caused by drugs or organic psychosis; (4) a history of neurological disorders (such as cerebral trauma, epilepsy, acute cerebrovascular disease etc.) or MRI evidence of structural brain abnormalities; (5) a history of significant medical illness (such as heart disease, hypertension, liver disease etc.), and a history of endocrine diseases (such as diabetes etc.), or other physical disease interfering with evaluation.; (6) current or past alcohol and drug abuse; (7) contraindications for undergoing an MRI scan, including metallic implants, retractors or braces, and claustrophobia; (8) not eating any functional food such as prebiotics or probiotics, nor did they administrate any antibiotics or other drugs influenced microbiota composition within 1 month prior to sampling; (9) no history of gastrointestinal disorders during the previous week.

Moreover, HCs without serious physical disorders matched for MDD group in age, years of education, marriage, and gender, were recruited from local communities through advertising. HCs were also assessed with the Mini-International Neuropsychiatric Interview (MINI) to ensure that they did not meet the criteria for any DSM-5 psychiatric disorder. Other exclusion criteria were the same with the MDD group.

All participants and their legal guardians voluntarily participated and signed an informed consent form before the experiment. Ethical approval was obtained from the local Ethics Committee at the First Affiliated Hospital, College of Medicine, Zhejiang University, China.

### Clinical assessments

Depressive symptoms and anxiety symptoms were assessed with HAMD-17 and Hamilton Anxiety Scale (HAMA), respectively. What’s more, the 30-item Inventory of Depressive Symptoms-Self Report (IDS-SR30) and the 16-item Quick Inventory of Depressive Symptomatology-Self Report (QIDS-SR16) were two self-report psychometric evaluations in patients with MDD. The two scales had proven useful as a sensitive way of determining a patient’s level of depression before treatment.

### Fecal samples collection and metagenomics sequencing analysis

Stool samples were collected on the same day of symptom assessment, frozen immediately, and stored at −80 °C before analyses. The stool samples were sent to the Novogene Bioinformatics Technology Co., Ltd. (Beijing, China) for sequencing. The NEBNext® Ultra™ DNA Library Prep Kit for Illumina (NEB, USA), the Illumina Novaseq 6000 platform, and Readfq version 8 (https://github.com/cjfields/readfq) was conducted to acquire the clean data. Then, the Clean Data is assembled and analyzed [25] by SOAPdenovo software version 2.04 (http://soap.genomics.org.cn/soapdenovo.html). After predicting the open reading frame by MetaGeneMark (V2.10, http://topaz.gatech.edu/GeneMark/) software, redundant genes were removed and obtain the unique initial gene catalog using CD-HIT [26, 27] version 4.5.8 (http://www.bioinformatics.org/cd-hit). Obtain the gene catalogue (Unigenes) by mapping and filtering genes. DIAMOND software (V0.9.9, https://github.com/bbuchfink/diamond/) is used to blast the Unigenes extracted from the NR database (Version: 2018-01-02, https://www.ncbi.nlm.nih.gov/) of NCBI. Choose the result of which the e value ≤ the smallest e value * 10 to take the LCA algorithm to make sure the species annotation information of sequences. The table containing the number of genes and the abundance information of each sample in each taxonomy hierarchy (kingdom, phylum, class, order, family, genus, species) are obtained based on the LCA annotation result and the gene abundance table.

The exhibition of generation situation of relative abundance, the exhibition of abundance cluster heat map, and PCA (R ade4 package, Version 2.15.3) analysis are based on the abundance table of each taxonomic hierarchy. Meta stats and the linear discriminant analysis effect size (LEfSe) analyses are used to look for different species between groups. LEfSe analysis is conducted by LEfSe software (the default LDA score is 2). Besides, random forest (RandoForest) (R pROC and randomForest packages, Version 2.15.3) was used to construct a random forest model. Screen out important species by Mean Decrease Accuracy, then cross-validate each model (default 10 times) and plot the ROC curve. Finally, predicted unigenes were used by DIAMOND Version 0.9.9 to assign to the KEGG. See supplementary 1.1 for detailed steps.

### Blood samples collection and metabolomic analysis

Blood was drawn immediately after symptom assessment and stored at −80 °C until assay. Blood samples were also sent to the Novogene Bioinformatics Technology Co., Ltd. (Beijing, China) for analysis. After pretreatment, they were injected into the HPLC-MS/MS system [28, 29] (SCIEX QTRAP^®^ 6500 + ) for subsequent analysis. LC-MS/MS analyses were performed using an ExionLC™ AD system (SCIEX) coupled with a QTRAP® 6500+ mass spectrometer (SCIEX). The processing parameters are in supplementary material 1.2. The detection of the experimental samples using MRM (Multiple Reaction Monitoring) was based on the Novogene Bioinformatics Technology Co., Ltd. (Beijing, China) in-house database. And the data files generated by HPLC-MS/MS were processed using the SCIEX OS Version 1.4 to integrate and correct the peak.

These metabolites were annotated using the KEGG database [30] (http://www.genome.jp/kegg/) and the HMDB database (http://www.hmdb.ca/). Partial least squares discriminant analysis (PLS‐DA) [31] was used to evaluate the difference in metabolic profiles between MDD and HC subjects that were performed at meta X version 1.4.16 [32]. Volcano plots were used to filter metabolites of interest. The metabolic pathway enrichment of differential metabolites was performed based on the KEGG database. See supplementary 1.2 for details.

### ELISA analysis

As stated above, blood samples were collected. Plasma samples were separated and stored at −80 °C for analysis after centrifuged at 3000 rpm for 20 min. Duoset human ELISA Kits (IL-1β: HSLB00D, R&D Systems; IL-6: HS60DC, R&D Systems; TNF: HSTA00E, R&D Systems) were used to respectively measure the plasma levels of the inflammatory factors, including IL-1β, IL-6, and tumor necrosis factor (TNF). According to the manufacturers’ instructions, the concentration of factors in each blood sample was quantitatively determined. All results were presented in pg/mL.

### MRI analysis

MRI images were scanned on a Philips Achieva3.0 T TX MRI system (Philips Healthcare, Netherlands). Resting-state-fMRI (rs-fMRI) data were acquired along the axial direction in a sequential mode using a fast field echo-echo- planar imaging (FFE-EPI) sequence: 24 slices, TR/ TE = 2000/35 ms, flip angle (FA) = 80°, slice thickness/gap = 5.0/1.0 mm, voxel size = 2.4 × 2.4 × 5.0 mm^3^, matrix = 100 × 100, field of view (FOV) = 240 ×;240 mm^2^.Meanwhile, the rs-fMRI scan lasted 6 min and 48 s. During the scanning, all participants were instructed to keep relaxed (eyes closed but awake) with the head placed in a head coil with foam to strengthening fixation.

The grey matter volume (GMV) was analyzed with Voxel-Based Morphometry (VBM) implemented in SPM8 software. T1-weighted images were processed by the Montreal Neurological Institute (MNI) template. First, the images were segmented into white matter, gray matter, and cerebrospinal fluid and bias correction was applied to remove image intensity non-uniformities. Then spatial registration was adopted for voxel-wise comparisons of GMV. During VBM analysis, the tissue volumes were reflected by the modulated images of the gray matter after bias correction. Finally, gray matter images were smoothed via a Gaussian filter with a full width at half maximum (FWHM) of 8 mm. Data of MRI were examined with two­sample t-tests, and the total intracranial volume, age, sex, and education years were set as a covariate.

### Statistics

SPSS (v. 25.0 Chicago, Illinois) software was used for statistical analysis of demographic, clinical, and immunologic information. All demographic and clinical variables were examined with two­sample t-tests, except sex. The sex data were analyzed with a χ^2^ test. Data of metagenomics, metabolomics, and MRI were analyzed as above. All data were presented as mean ± standard deviation for normally distributed continuous variables or median ± interquartile range for non-normal distributed continuous variables. Besides, all statistical tests were two­tailed.

To determine the association in gut microbiota, metabolites, inflammatory cytokines, and grey matter volume in MDD patients, we constructed a heatmap about correlation analysis using Pearson’s correlations in R version 3.4.3 (Psych package). The Pearson correlation index was calculated and tested for significance by the Corr. test function. Then the Pheatmap function in the Pheatmap package is used for visualization. Taking the absolute value of *r* ≥ 0.3, which is considered to be correlated. For all the data, a P-value < 0.05 was considered to be statistically significant.

## Result

### Clinical characteristics of the participants

A total of 30 MDD subjects and 30 HCs were recruited. Three MDD patients and three HCs were excluded due to excessive motion when MRI Scanning. There were also four subjects removed. One healthy subject was due to the low fecal volume, and three patients were because the blood samples did not pass quality detection. Therefore, our samples ultimately consisted of 24 MDD patients and 26 controls.

The detailed information including demographic and clinical characteristics of participants was presented in Table 1. The HAMD-17, HAMA, IDS-SR30, and QIDS-SR16 scores showed a significant difference between the MDD and HCs (all *p* < 0.001). There was no significant difference between the two groups in age, gender, or years of education.

**Table 1:** Detailed clinical characteristics of the participants.

Variables	MDD (*n* = 24)	HC(*n* = 26)	*t*/*χ*^*2*^	*p*
Course, (month)	7.50 ± 5.45	–	–	–
Age, (year)	29.96 ± 8.554	31.31 ± 9.707	−0.520^a^	0.606
Sex (male/female)	7/17	8/18	0.015^b^	0.902
Education years	14.25 ± 2.382	14.04 ± 3.092	0.269^a^	0.789
HAMD-17	24.83 ± 3.116	2.12 ± 2.776	27.262^a^	<0.001*
HAMA	19.33 ± 4.622	1.31 ± 2.655	17.059^a^	<0.001*
IDS-SR_30_	21.79 ± 8.885	1.81 ± 2.668	10.593^a^	<0.001*
QIDS-SR_16_	18.54 ± 6.057	1.73 ± 2.616	12.915^a^	<0.001*

^a^Two‐tailed student’s t-test for continuous variables;

^b^Chi‐square analyses for categorical variables (sex); *P < 0.05.

### Alterations of gut microbiota in MDD patients

**Fig. 1:**
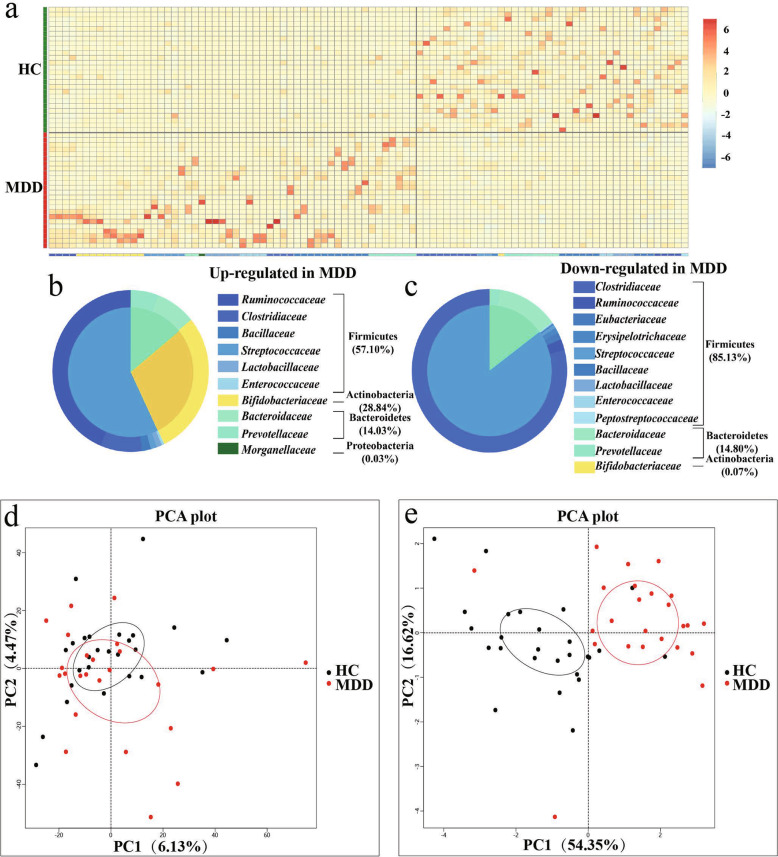
Alterations of gut microbiota in MDD patients. **a**–**c** Using meta-stat analysis, 94 differential species responsible for discriminating the gut microbiota in MDD and HCs subjects were identified. **a** Heatmap of the 94 differential species abundances between MDD subjects and HCs. **b** 54 upregulated species in MDD are arranged on the left, while 40 decreased species are arranged on the right. **c** Most of the upregulated species belong to Firmicutes (57.10%), Actinobacteria (28.84%), and Bacteroidetes (14.03%), while downregulated species mainly belong to Firmicutes (85.13%) and Bacteroidetes (14.80%). **d** Principal component analysis (PCA) showed that gut microbial composition of MDD patients was significantly different from that in HCs at the species level. (*n* = 24, MDD group; n = 26, HC group). **e** Principal component analysis (PCA) revealed the differences in microbial functions between the MDD patients and HCs on KEGG level 3.

**Fig. 2:**
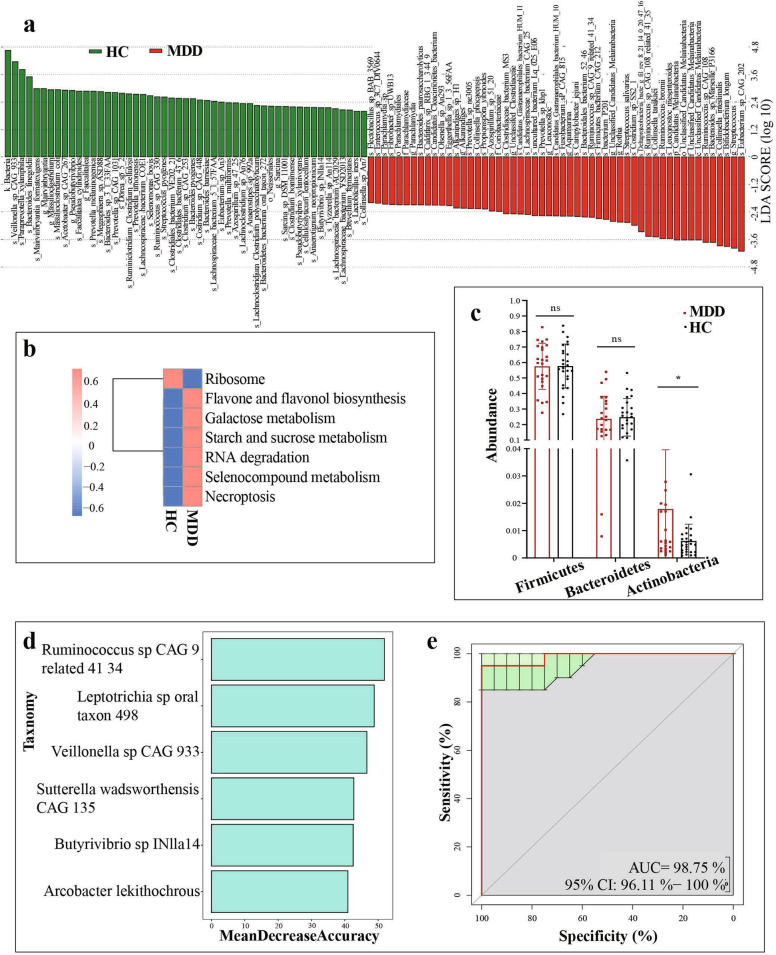
Alterations of gut microbiota in MDD patients. **a** LDA scores revealed different composition in bacterial taxa between MDD and HCs (LDA = 2). Red bars indicate taxa were enrichment in MDD, and green bars represent abundant bacterial taxa in HCs. **b** Differential biological processes of gut microbiome function in MDD groups (red) relative to HCs (blue). **c** At the phylum level, the abundance of Firmicutes and Bacteroidetes were decreased in the MDD group relative to HCs, while Actinobacteria were significantly higher in the MDD group than in the HC group. ns: *p* > 0.05; *p < 0.05. **d**–**e** Using random forest models to predicted MDD diagnosis through biomarkers. **b** Six Biomarkers were screened according to Mean Decrease Accuracy in random forest. Rank the species from top to bottom according to their degree of contribution. **c** Receiver operating characteristic (ROC) curves of response predicted by random forest models. The area under the ROC curve (AUC) was 0.98 (95% CI: 0.961–1).

The principal component analysis (PCA) revealed striking differences in microbial composition between the MDD and HC groups at the species level (Fig. 1(d)). A characteristic alteration of the microbiota was predicted by the linear discriminant analysis (LDA), which removed the species belonging to the virus Kingdom. LDA showed that Ruminococcaceae, Parachlamydiaceae, and Coriobacteriaceae families had a higher level in MDD patients compared with HCs, while the families Lachnospiraceae, Clostridiaceae, Prevotellaceae, and Bacteroidaceae were more abundant in HCs (LAD score = 2; Fig. 2(a)).

Subsequently, 94 differential gut microbiotas were identified by meta stat analysis, which represented the bacterial profile difference between the MDD and HC groups (Fig. 1(a)). Consistent with the LDA results, Ruminococcaceae was increased in the MDD group, while Clostridiaceae, Bacteroidaceae, and Prevotellaceae were decreased. More precisely, relative to HCs, the 40 species that were decreased in MDD groups mainly belonged to the bacterial taxonomic families Clostridiaceae (9 species), Bacillaceae (6 species), Ruminococcaceae (4 species), Bacteroidaceae (4 species), and Prevotellaceae (4 species) (Fig. 1(c)). What’s more, the MDD groups were characterized by 54 increased species (Fig. 1(b)), which mainly belonged to the bacterial families Bacillaceae (11 species), Bifidobacteriaceae (10 species), Prevotellaceae (7 species), Streptococcaceae (6 species), Lactobacillaceae (5 species), Ruminococcaceae (4 species), and Clostridiaceae (4 species). Overall, the 94 discriminative species primarily belonged to the phyla Firmicutes (65/94, 69.15%), Bacteroidetes (17/94, 18.09%), and Actinobacteria (11/94, 11.70%). Furthermore, Firmicutes and Bacteroidetes (both *p* > 0.05) had no statistically significant difference in the MDD group, while Actinobacteria (*p* < 0.05) were higher in the MDD group than in the HC group at the phylum level (Fig. 2(c)).

To predict biomarkers of MDD, random forest classification models were constructed with ten-fold cross-validation at the species level (Fig. 2(d)). It was assessed using a receiver operating characteristic (ROC) curve. According to the number of biomarkers and the area under the ROC curve (AUC), we chose the best model which contained six species: Ruminococcus sp CAG: 9 related 41 34, Leptotrichia sp oral taxon 498, Veillonella sp CAG: 933, Sutterella wadsworthensis CAG: 135, Butyrivibrio sp INlla14, Arcobacter lekithochrous. The AUC was 0.98 (95% CI: 0.961–1) (Fig. 2(e)).

Additionally, metagenomic sequencing allows for the analysis of gut microbial functions. PCA based on KEGG level 3 revealed differences in microbial functions between the MDD and HCs (Fig. 1(e)). Here, we identified a total of 293 differential Kyoto Encyclopedia of Genes and Genomes (KEGG) orthology genes (KO genes) between the two groups, which were mainly involved in seven biological processes (especially four metabolic pathways) (Fig. 2(b)). The abundance of KO gene copies associated with galactose metabolism, starch and sucrose metabolism, selenocompound metabolism, and flavone and flavonol biosynthesis were increased in the MDD patients relative to HCs.

### Metabolites showing a significant difference between MDD and HCs

**Fig. 3:**
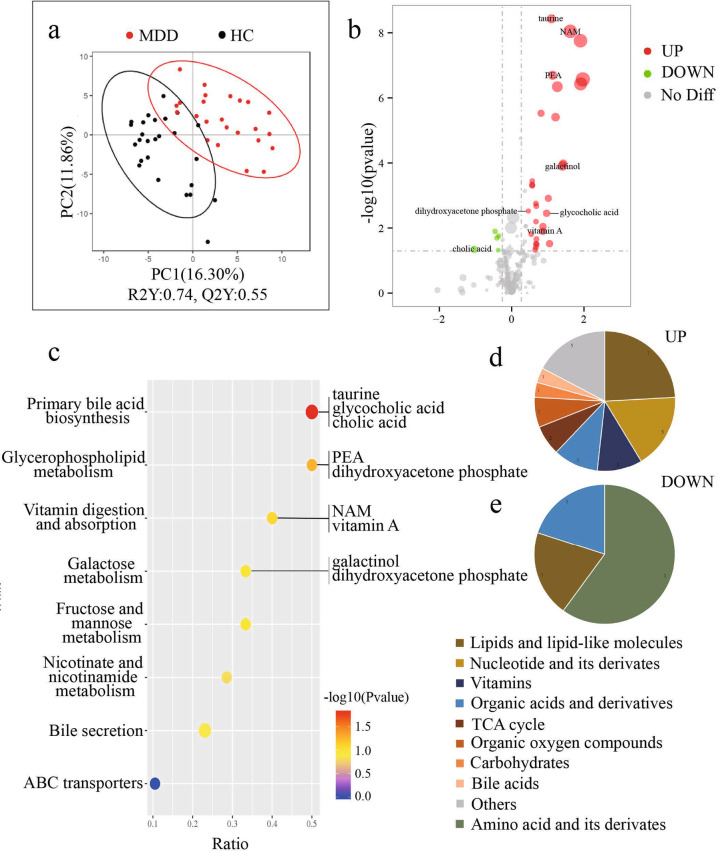
Metabolites showing a significant difference between MDD and HCs. **a** Partial least squares discriminant analysis (PLS-DA) establishes the model by partial least square regression showing clear discrimination between MDD and HCs. **b** The volcanic map shows 34 significantly different metabolites. Red dots represent increased metabolites; while green dots indicate decreased metabolites. The size of the dot represents the Variable Importance for the Projection (VIP). Vertical coordinate shows levels of significance. **c** The bubble chart shows KEGG pathways between two groups, which excludes the pathway which annotates only one metabolite. The size of the dot represents the number of metabolites in the pathway. The red dots are more significant than the blue dots. The metabolites of the top three pathways were labeled both in figs. **b** and **c**. **d–e** A total of 29 increased metabolites in the MDD group are arranged on the left, while 5 downregulated metabolites are arranged on the right. Most of the upregulated metabolites belong to lipids and lipid-like molecules (24.14%), nucleotide and its derivates (17.24%), vitamins (10.34%), and organic acids and derivatives (10.34%); while downregulated metabolites mainly belong to amino acid and its derivatives (60.00%).NAM Nicotinamide, PEA Phosphoethanolamine.

Metabolites from MDD and HCs were used for partial least squares discriminant analysis (PLS-DA), which showed distinct separation between two groups (Fig. 3(a)). It suggests that MDD patients have a dissimilar metabolic mode relative to HCs. Compared to the HC group, a total of 34 significantly different metabolites were identified, with 29 increased and 5 decreased in the MDD group (Fig. 3(b)). The top six metabolites by P-value are taurine, nicotinamide (NAM), 3’-aenylic acid, phosphoethanolamine (PEA), adenosine 5’-monophosphate, and dl-dihydrosphingosine. According to HMDB classification, 29 increased metabolites mainly belong to lipids and lipid-like molecules (7/29, 24.14%), nucleotide and its derivates (5/29, 17.24%), vitamins (3/29, 10.34%), organic acids and derivatives (3/29, 10.34%) (Fig. 3(d)). In addition, amino acid and its derivatives (3/5, 60.00%), lipids and lipid-like molecules (1/5, 20.00%) and organic acids and derivatives (1/5, 20.00%) were the metabolites decreased in the MDD group (Fig. 3(e)).

We identified forty discernible KEGG pathways between the two groups. The result excluding the pathway which annotates only one metabolite was shown in Fig. 3(c). The top four pathways based on P-value were the primary bile acid biosynthesis, glycerophospholipid metabolism, vitamin digestion and absorption, and galactose metabolism. They are mainly involved in lipid, vitamin, and carbohydrate metabolism. The differential metabolites participating in the four pathways have been labeled in Fig. 3 (b) and (e), namely taurine, NAM, PEA, galactinol, dihydroxyacetone phosphate, glycocholic acid, vitamin A, and cholic acid. Meanwhile, analysis of gut microbial functions showed that patients with MDD were mainly characterized by disturbances of carbohydrates. Integration of these findings showed that disturbance of carbohydrates metabolism, especially galactose metabolism, may be particularly relevant to the gut ecosystem of MDD.

### Plasma inflammatory factors level in MDD patients versus HCs

**Fig. 4:**
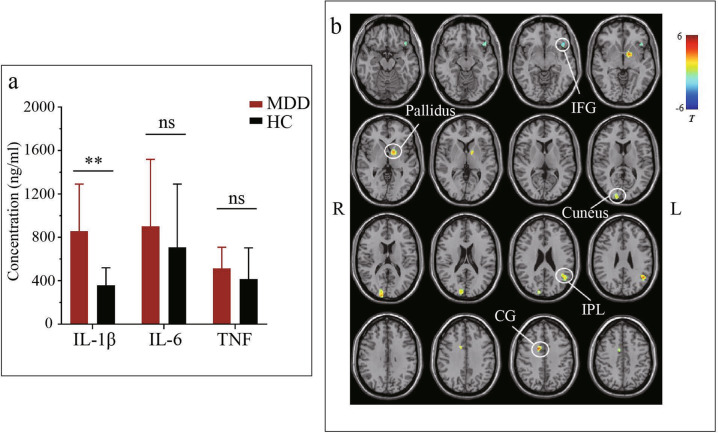
Differences of plasma levels of inflammatory factor and Grey matter volume between MDD and HCs. **a** The data indicate that patients with MDD have significantly higher IL-1β levels (858.30 ± 432.70 pg/ml) than HCs (359.52 ± 160.63 pg/ml). However, the MDD patients showed no significant change in IL-6 and TNF compared with HCs (901.03 ± 617.92 pg/ml vs. 707.31 ± 584.07 pg/ml, 514.12 ± 194.41 pg/ml vs. 414.87 ± 288.36 pg/ml, respectively). ns: *p* > 0.05, **p < 0.001. **b** The cold color indicates decreased volume, and the warm color represents the increased volume in MDD compared with HCs. IFG Inferior frontal gyrus, IPL Inferior parietal lobe, CG Cingulate gyrus.

Increased IL-1β plasma level was observed in MDD patients (858.30 ± 432.70 pg/ml vs 359.52 ± 160.63 pg/ml, P < 0.001, Fig. 4(a)). Nevertheless, the MDD patients showed no statistically significant difference in IL-6 and TNF concentration compared with HCs (901.03 ± 617.92 pg/ml vs 707.31 ± 584.07 pg/ml, *P* = 0.265; 514.12 ± 194.41 pg/ml vs. 414.87 ± 288.36 pg/ml, *P* = 0.166, respectively, Fig. 4).

### GMV results

Compare to HC subjects, MDD patients showed significantly decreased GMV in the left inferior frontal gyrus (IFG), and increased GMV in the left pallidum, left cuneus, left inferior parietal lobe (IPL), and right cingulate gyrus (CG) (Fig. 4(b)).

### Correlations between gut microbiome and metabolites, IL-1β, GMV, clinical characteristics of MDD

**Fig. 5:**
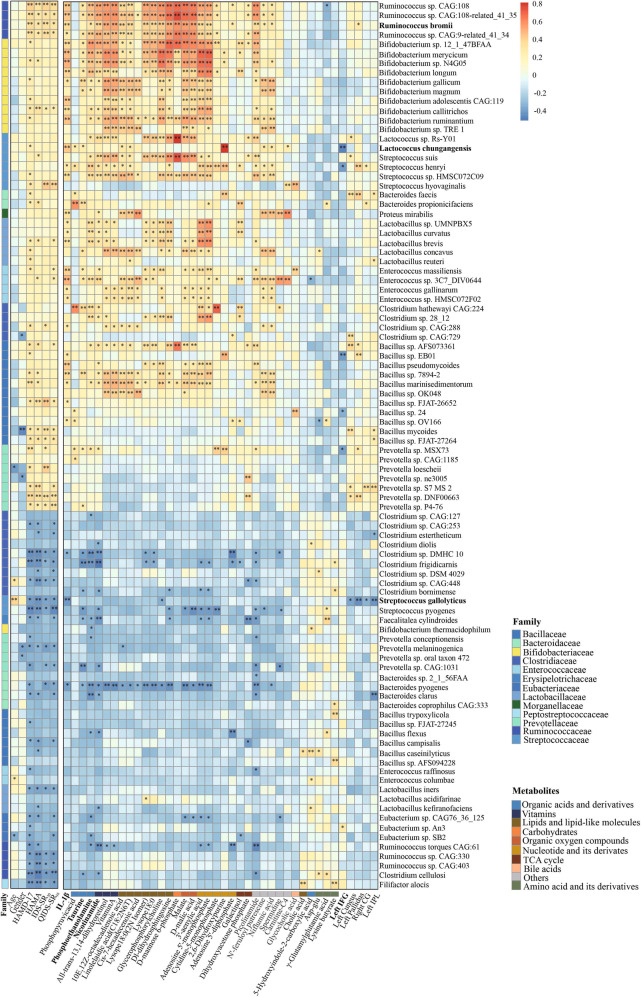
Correlations between gut microbiome and metabolites, IL-1β, GMV, clinical characteristics of MDD. Heat map of the Pearson correlation coefficient of 94 species of the gut microbiome, IL-1β, 34 metabolites, and 5 brain regions as well as 6 clinical indexes. The X-axis is clinical indexes (including age, gender, and clinical scales), IL-1β, metabolites (consisted mainly of organic acids and derivatives, vitamins, lipids, and lipid-like molecules, etc.), and GMV of brain regions, in order. Y-axis is the 94 species of the gut microbiome. Red squares indicate positive associations; while blue squares indicate negative associations. The statistical significance was denoted on the squares (*p < 0.05, **p < 0.01). HAMD-17 The 17-item Hamilton Depression scale, HAMA Hamilton Anxiety Scale, IDS-SR_30_ 30-item Inventory of Depressive Symptoms-Self Report, QIDS-SR_16_ 16-item Quick Inventory of Depressive Symptomatology-Self Report, IFG Inferior frontal gyrus, IPL Inferior parietal lobe, CG Cingulate gyrus.

To further explore the relationships between disturbances of the gut microbiome, metabolome, immunology, brain structure, and MDD clinical characteristics, a correlation heat map was generated using Pearson correlation (Fig. 5). The X-axis was 6 clinical indexes, IL-1β, 34 metabolites, and GMV of 5 brain regions, in order. Y-axis was the 94 species of the gut microbiome.

To our delight, half of the gut microbiomes (Ruminococcus being the most important) were highly linked with clinical parameters, such as HAMD-17, HAMA, IDS-SR_30_, and QIDS-SR_16_. Additionally, the matrix mainly showed a strong positive association between up-regulated microbiota in the MDD group and IL-1β, which include Ruminococcus, Bifidobacterium, Enterococcus, Proteus. Our results proved that differential gut microbiota was generally relevant to differential metabolites [4, 33]. We also found the high abundance of bacteria (mainly containing Ruminococcus and Bifidobacterium) in MDD showed significant positive correlations with the up-regulated metabolites except for organic oxygen compounds, carbohydrates, and bile acids. In the low abundance of bacteria in MDD, Clostridium, Streptococcus, and Bacteroides were negatively associated with organic acids and derivatives, vitamins, nucleotide and its derivates, and Lipids and lipid-like molecules, which increased in the MDD group, while Bacillus and Filifactor had a positive connection with decreased metabolites (amino acid and its derivatives) in MDD group. Besides, the organic oxygen compounds which were involved in galactose metabolism were positively associated with the abundance of some Bifidobacterium, Streptococcus, Lactococcus, and Prevotella.

Although the correlation between the gut microbiome and GMV was relatively weak, it can be seen that the rising microbiota (Bacteroides and Prevotella) and the descending microbiota Streptococcus were positively or negatively related to left pallidum, left cuneus, left IPL, and right CG, respectively. These brain regions’ GMV was increased in MDD patients. What’s more, rising Lactococcus, Streptococcus, and Bacillus were negatively associated with left IFG, while declining microbiota Eubacterium showed positive relation.

Most notably, Ruminococcus bromii, Lactococcus chungangensis, and Streptococcus gallolyticus were associated with metabolome, immunology, brain structure, and clinical scales. All three microbiota were linked to IL-1β. In addition, Ruminococcus bromii had a positive correlation with the concentration of PEA, NAM, all-trans-13,14-dihydroretinol, vitamin A, 10E,12Z-octadecadienoic acid, linolelaidic acid (C18:2 N6T), lysope18:0(2N Isomer), lysope18:0, glycerophosphorylcholine, dl-dihydrosphingosine, d-mannose 6-phosphate, malate, D-malic acid and the GMV of left pallidum. Lactococcus chungangensis was positively related to phosphopyruvicacid, 10E,12Z-octadecadienoic acid, linolelaidic acid (C18:2N6T), and 2,6-Dihydroxypurine, while was negatively related to the GMV of left IFG. Streptococcus gallolyticus was negatively connected with glycerophosphorylcholine, the GMV of left cuneus, left pallidum, and left IPL.

## Discussion

This is the first time that the multi-level effects of gut microbiota have been studied in MDD patients. We supposed that the gut microbiome exerts its effects in MDD is multifactorial, such as lipid and vitamin metabolism disorder, proinflammatory effect and modulate structure, and function of the brain. A preclinical study confirmed that the gut microbiome of CUMS rats leads to the disorder of liver metabolism and inflammation in the brain by disrupting the intestinal barrier. [34] In our result, it is noteworthy that Ruminococcus bromii, Lactococcus chungangensis, and Streptococcus gallolyticus were widely related to metabolic disorders, IL-1β, GMV, and clinical scales, which provide clues for us to find the interaction of each omics.

More and more evidence has shown a strong association between MDD and the gut microbiome. In our study, gut microbiota compositions in drug-naïve MDD patients were dominantly characterized by Actinobacteria, which was consistent with most previous studies [11–13]. Besides, consistent with P Zheng et al. [11] and Kelly JR et al. [17], we observed that Firmicutes and Bacteroidetes had no statistically significant difference between MDD patients and HCs. At the family level, we found Ruminococcaceae, Streptococcaceae, Lactobacillaceae, Clostridiales, and Bifidobacteriaceae were increased, while Bacteroidaceae, Lachnospiraceae, and Prevotellaceae were decreased in MDD groups compared with HCs. P Zheng et al. [11] also showed that Ruminococcaceae, Streptococcaceae, Lactobacillaceae, and Clostridiales were overrepresented in MDD subjects, while Bacteroidaceae and Lachnospiraceae were overrepresented in HCs. The study of Yu-Chu Ella Chung et al. [13] reported that Streptococcaceae and Bifidobacteriaceae increased, while Prevotellaceae decreased in MDD groups. Studies of metagenomics often show some inconsistencies with previous reports [14]. Overall, however, the microbial signatures of MDD patients in the present study are consistent with previous studies.

All three species of gut microbiota mentioned above were linked to IL-1β and lipids. Increased IL-1β plasma level was observed in MDD patients, which has been widely confirmed [35–40]. Our results also demonstrated that lipids (phosphatidylethanolamine (PE) [41], polyunsaturated fatty acids [42, 43]), as well as organic acids, which was involved in lipid metabolism (taurine [44], PEA [45]) are the key to distinguish MDD from HCs. PEA and taurine were involved in important cellular functions, including neuromodulation and membrane stabilization [46]. As a precursor for PE, PEA has been considered to play a crucial role in myelination [47]. Recent research reported that the elevated PEA level could reflect cerebral phospholipid turnover, which is an indicator of neural membrane synthesis and signal transduction [48]. It was reported that socially withdrawn mice had impaired myelination in the prefrontal cortex (PFC). [49] Taurine also has membrane-stabilizing effects on the CNS. Moreover, there is an interrelationship between extracellular taurine and PEA [50]. Therefore, we speculated that PEA and taurine might involve in MDD by affecting nerve myelination. Interestingly, PE has been identified as an inflammatory modulator [51]. Li B et al. [52] suggested that liver glycerophospholipid metabolism disorder indicated oxidative stress, inflammatory cell membrane damage, and even apoptosis in mice transplanted feces from MDD patients.

Previous studies have explored the mechanism of the MGB axis about immunology and metabolomics respectively. However, few studies have explored the correlation between gut microbiota, metabolomics, immunology, and brain regions. By combining the four omics data, our research found that Ruminococcus bromii and Lactococcus chungangensis had a positive correlation with the concentration of IL-1β, 10E,12Z-octadecadienoic acid, and linolelaidic acid (C18:2N6T). Ruminococcus bromii and Streptococcus gallolyticus were positively or negatively connected with glycerophosphorylcholine, respectively. Research shows NOD-like receptor family pyrin domain containing 3(NLRP3) inflammasome has been considered as a link between lipid metabolism and inflammation [53]. 10E,12Z-octadecadienoic acid and linolelaidic acid (C18:2N6T) are polyunsaturated fatty acids (PUFAs). A review concludes that PUFAs have recently been shown to impede NLRP3 activity [54]. Glycerophosphorylcholine is a component of phosphatidylcholine (PC). A study made by Yeon SH et al. [55] demonstrated that lipids such as oxidized PC induce the activation of the NLRP3 inflammasome, leading to the production of IL-1β. A study in gouty nephropathy patients shows that increased lipids, in particular the lysophosphatidylethanolamine (LPE) and PC, could activate the NLRP3 inflammasome [56]. In addition, LPE is the product of hydrolyzed PE, whose precursor is PEA. Ruminococcus bromii had a positive correlation with the concentration of PEA. previous studies suggested that NLRP3 inflammasome mediates the level of IL1β in the PFC that results in depressive-like behavior after stress. [57–59] Specifically, psychosocial stress-induced damage-associated molecular patterns (DAMPs), such as bacteria and bacterial products, which leaked from the gut into the periphery. These DAMPs subsequently activated inflammatory signaling pathways, especially NLRP3 inflammasome. Stimulation of NLRP3, in turn, activates caspase 1, leading to the production of mature IL-1β and IL-18, which enter the brain through humoral and neural routes, causing central inflammatory [60]. Furthermore, previous studies show that pro-inflammatory cytokines in peripheral blood are associated with the reduction of gray matter volumes, such as hippocamp and CG [61, 62]. In the present study, Lactococcus chungangensis was negatively correlated with the GMV of IFG. We further investigated the relationship between depressive severity and gut microbiota. Consistent with previous studies [12, 63], our results showed that the abundance of Ruminococcaceae, Faecalibacterium, Clostridium, and Streptococcus were negatively related to total scores of HAMA, HAMD-17, IDS-SR_30_, and QIDS-SR_16_. Therefore, we came up with the hypothesis that Ruminococcus bromii may involve in the pathogenesis of MDD by causing the lipid disturbance (especially PEA and glycerophosphorylcholine), and activating the NLRP3 inflammasome in IFG. These results are preliminary and require further validation. Besides, Streptococcus gallolyticus was negatively connected with IL-1β and glycerophosphorylcholine, so it may play a probiotic role to impede NLRP3 activity in MDD.

However, to our confusion, previous studies showed that Ruminococcus bromii [64] and Lactococcus chungangensis [65] have a probiotics-like effect, while they have a positive correlation with IL-1β in MDD patients in the present study. Furthermore, Lactococcus chungangensis was positively connected with PUFAs (10E,12Z-octadecadienoic acid and linolelaidic acid (C18:2N6T)), that has been shown to impede NLRP3 activity. On the premise of ensuring our experimental procedures are rigorous, we speculated the reasons for this phenomenon. The MDD subjects we recruited were drug-naïve patients whose homeostasis may be maintained through a compensatory mechanism. We thought the depressed individuals might alter metabolism and immune activity, which in turn was antagonized by gut microbiota. [6] But our study can’t clarify whether the gut microbiota is the cause or consequence of depression.

Besides, we agree with the results of Chen JJ et al. [66, 67], which suggests that vitamins, especially NAM, were involved in the development of MDD as well. Combining gut microbial functions and metabolomics analysis, showed that disturbance of galactose metabolism was relevant to the MDD. P Zheng et al. [11] also reported that the abundance of gene copies associated with carbohydrate metabolism was increased in MDD patients. A detailed discussion can be seen in supplement 2.

The present study may be limited by a relatively small sample size, because it is difficult to collect MRI data and fecal and blood samples for each MDD patient. This may limit not only the statistical validity assessment but also the subcategories analysis of depression, which restricts the generalization and precision of our findings. Second, effects of diet [68] and regional [69] variation in the composition of gut microbiota were inevitable. In our study, using antibiotics, probiotics, and prebiotics was not allowed. The participants’ diets were similar since they are of Han ethnicity. But we did not record the information about detailed diets, such as food type, caloricity and cooking style, etc. Similarly, diet also has an impact on the plasma levels of metabolites. [70] Third, we did not collect the data after treatment. Dynamically comparing changes in data before and after treatment may be more helpful in the future. Fourth, we did not verify our hypothesis in the animal experiment. Therefore, a larger sample size and multiregional cooperation study are required in future studies, which can contribute to a better understanding of the mechanism of gut microbiota in MDD. Meanwhile, carrying out researches between the subcategories of depression and gut microbiota would be of more clinical significance.

Overall, based on multi-omics data, we demonstrated that the effects of gut microbiome exert in MDD is multifactorial. The alteration of gut microbiota was associated with metabolism disorder, immune activation, and changed GMV in the brain. It is speculated that the NLRP3 inflammasome plays an important role in the MGB axis, as a link between lipid metabolism and inflammation. These findings provided a novel insight into the pathologic mechanisms underlying depression. This pilot analysis of multi-omics was helpful for future investigations to develop diagnostic or therapeutic tools of MDD.

## Supplementary information

Supplemental Material

## Data Availability

The raw data supporting the conclusions of this article will be made available by the authors, without undue reservation.
