# Supplementary material for: A pilot exploration of multi-omics research of gut microbiome in major depressive disorders
Source: Transl Psychiatry. 2022 Jan 10;12:8. doi: 10.1038/s41398-021-01769-x (PMC8748871; doi:10.1038/s41398-021-01769-x)
Supplement: Supplementary file 1 — Supplemental Material [file 41398_2021_1769_MOESM1_ESM.docx]

**1.** **Methods**

**1.1Metagenomics sequencing analysis**

Stool samples were collected on the same day of symptom assessment, frozen immediately, and stored at −80 °C before analyses. Bacterial genomic DNA was extracted according to the standard Stool Extraction Kit protocol after thawed. A total amount of 1μg DNA per sample was used as input material for the DNA sample preparations.

Sequencing libraries were generated using NEBNext® Ultra™ DNA Library Prep Kit for Illumina (NEB, USA) following the manufacturer’s recommendations and index codes were added to attribute sequences to each sample. Then, the libraries were sequenced on the Illumina Novaseq 6000 platform and paired-end reads were generated. Readfq version 8 (https://github.com/cjfields/readfq) was conducted to acquire the clean data at the Novogene Bioinformatics Technology Co., Ltd. (Beijing, China).

The Clean Data is assembled and analyzed^1^ by SOAPdenovo software version 2.04 (<http://soap.genomics.org.cn/soapdenovo.html>). After predicting the open reading frame by MetaGeneMark (V2.10, http://topaz.gatech.edu/GeneMark/) software, redundant genes were removed and obtain the unique initial gene catalog using CD-HIT^2,3^ version 4.5.8 (http://www.bioinformatics.org/cd-hit). Next，the Clean Data of each sample is mapped to the initial gene catalog using Bowtie2.2.4 and get the number of reads to which genes mapped in each sample, after filtering the gene which the number of reads ≤ 2 in each sample and obtain the gene catalogue (Unigenes) eventually used for subsequent analysis. ) Based on the number of mapped reads and the length of the gene, a statistic the abundance information of each gene in each sample.

DIAMOND software (V0.9.9, https://github.com/bbuchfink/diamond/) is used to blast the Unigenes to the sequences of Bacteria, Fungi, Archaea and Viruses which are all extracted from the NR database (Version: 2018-01-02, https://www.ncbi.nlm.nih.gov/) of NCBI. For the finally aligned results of each sequence, as each sequence may have multiple aligned results, choose the result of which the e value ≤ the smallest e value * 10 to take the LCA algorithm which is applied to system classification of MEGAN software to make sure the species annotation information of sequences. The table containing the number of genes and the abundance information of each sample in each taxonomy hierarchy (kingdom, phylum, class, order, family, genus, species) are obtained based on the LCA annotation result and the gene abundance table. The abundance of a specie in one sample equals the sum of the gene abundance annotated for the specie; the gene number of a specie in a sample equals the number of genes whose abundance is nonzero.

The exhibition of generation situation of relative abundance, the exhibition of abundance cluster heat map, and PCA (R ade4 package, Version 2.15.3) analysis are based on the abundance table of each taxonomic hierarchy. Meta stats and the linear discriminant analysis effect size (LEfSe) analyses are used to look for different species between groups. Permutation test between groups is used in Meta stats analysis for each taxonomy and get the P-value, then use Benjamini and Hochberg False Discovery Rate to correct P-value and acquire q value. LEfSe analysis is conducted by LEfSe software (the default LDA score is 2). Besides, random forest (RandoForest) (R pROC and randomForest packages, Version 2.15.3) was used to construct a random forest model. Screen out important species by Mean Decrease Accuracy, then cross-validate each model (default 10 times) and plot the ROC curve. Finally, predicted unigenes were used by DIAMOND Version 0.9.9 to assign to the KEGG. The abundances of each functional annotation were the sum of the abundance of annotation of each functional level.

**1.2 Metabolomic analysis**

Blood was drawn from the antecubital fossa immediately after symptom assessment. Then, samples were stored at −80 °C until assay. Blood samples (100 μL) and prechilled methanol (400 μL) were mixed by good vortexing. The samples were incubated on ice for 5 min and then were centrifuged at 15000 rpm, 4°C for 10 min. Some of the supernatants were diluted to the final concentration containing 60% methanol by HPLC-MS grade water. The samples were subsequently transferred to a fresh Eppendorf tube with 0.22 μm filter and then were centrifuged at 15000 g, 4°C for 10 min. Finally, the filtrate was injected into the HPLC-MS/MS system^4,5^ (SCIEX QTRAP^®^ 6500+) analysis.

LC-MS/MS analyses were performed using an ExionLC™ AD system (SCIEX) coupled with a QTRAP® 6500+ mass spectrometer (SCIEX). Samples were injected onto a BEH C8 Column (100×2.1 mm, 1.9μm) using a 30-min linear gradient at a flow rate of 0.35mL/min for the positive polarity mode. Negative Ion Mode Samples were injected onto aHSS T3 Column (100 mm×2.1 mm) using a 25-min linear gradient at a flow rate of 0.35mL/min for the negative polarity mode. The eluents were eluent A (0.1% Formic acid-water) and eluent B (0.1%Formic acid-acetonitrile).The solvent gradient for the positive polarity mode was set as follows: 5% B, 1 min; 5-100% B, 24.0 min; 100% B, 28.0 min; 100-5% B, 28.1 min; 5% B, 30 min. However, for the negative polarity mode, gradient was set as follows: 2% B, 1 min; 2-100% B, 18.0 min; 100% B, 22.0 min；100-5% B, 22.1 min；5% B, 25 min. QTRAP® 6500+ mass spectrometer was operated in positive polarity mode with Curtain Gas of 35 psi, Collision Gas of Medium, IonSpray Voltage of 5500V, Temperature of 500℃, Ion Source Gas of 1: 55, Ion Source Gas of 2: 55. The parameters of negative polarity mode were consistent with the positive polarity mode except that the IonSpray Voltage is -4500V.

The detection of the experimental samples using MRM (Multiple Reaction Monitoring) was based on the Novogene Bioinformatics Technology Co., Ltd. (Beijing, China) in-house database. The Q3 was used for metabolite quantification. The Q1, Q3, RT (retention time), DP (declustering potential), and CE (collision energy) were used for the metabolite identification. The data files generated by HPLC-MS/MS were processed using the SCIEX OS Version 1.4 to integrate and correct the peak. The main parameters were set as follows: minimum peak height, 500; signal/noise ratio, 10; gaussian smooth width, 3. The area of each peak represents the relative content of the corresponding substance.

These metabolites were annotated using the KEGG database^6^ (http://www.genome.jp/kegg/) and HMDB database (http://www.hmdb.ca/). Partial least squares discriminant analysis (PLS‐DA)^7^ was used to evaluate the difference in metabolic profiles between MDD and HC subjects that were performed at meta X version 1.4.16^8^. We applied univariate analysis (t-test) to calculate the statistical significance (P-value). The metabolites with the variable importance for the projection (VIP) > 1 and P-value< 0.05 and fold change≥2 or FC ≤0.5 were considered to be differential metabolites. Based on Log2(FC) and -log10(P-value) of metabolites, volcano plots were used to filter metabolites of interest. The functions of these metabolites and metabolic pathways were studied using the KEGG database. The metabolic pathway enrichment of differential metabolites was performed, when the ratio was satisfied by x/n > y/N, the metabolic pathway was considered as enrichment, when the P-value of metabolic pathway < 0.05, the metabolic pathway was considered as statistically significant enrichment.

**2. Discussion**

Besides, we agree with the results of Chen JJ et al.^9,10^, which suggests that vitamins, especially NAM, were involved in the development of MDD as well. As we know, NAM was involved in both vitamin metabolism and the tryptophan–nicotinic acid pathway, meanwhile, the tryptophan was a biochemical precursor of serotonin^11,12^. Previous studies have suggested that altered serotonergic neurotransmission may contribute to the etiology of depression^13,14^. Therefore, we speculated that the variation of NAM might indicate serotonin biosynthesis disorders in depression patients. Moreover, Blacher E et al. indicated that in Amyotrophic lateral sclerosis (ALS) mice, Ruminococcus exacerbate the symptoms of ALS, while the supplement of NAM can improve motor symptoms.^15^ This study also found Ruminococcus bromii was positively associated with NAM, IL-1β, and volume of pallidum. NAM was the major precursor of coenzyme NADH/NAD+.^16^ As a sirtuin 1 (SIRT1) agonist, NAD+ may activate SIRT1 by regulating the ratio of NAD+/NADH.^17^ SIRT1 could positively modulate the regulator of NF-κB pathway, which leads to an increase in peripheral pro-inflammatory cytokines.^18^ Meanwhile, the activated microglia, mediated by NAM adenine dinucleotide phosphate (NADPH) oxidases, secreted neurotoxic molecules such as several chemokines and proinflammatory cytokines IL-6, TNF, and IL-1β, which had harmful effects on the blood-brain barrier.^19^ As mentioned above, pro-inflammatory cytokines are associated with gray matter volumes^20,21^. We suspect that Ruminococcus bromii may activate microglia mediated by NAM disorder and cause inflammation, which ultimately leads to the change of GMV in thestriatum in MDD.

Additionally, combining gut microbial functions and metabolomics analysis, showed that disturbance of galactose metabolism was relevant to the MDD. P Zheng et al.^22^ also reported that the abundance of gene copies associated with carbohydrate metabolism was increased in MDD patients. In the study of MDD rats, Wei-Jie Lv et al.^23^ suggested carbohydrate metabolism was significantly changed. Here, we reported that galactose metabolism within carbohydrate metabolism may be a new disturbing pathway between the MDD and HC groups.

**Reference**

1 Luo, R., Liu, B., Xie, Y., Li, Z., Huang, W., Yuan, J. *et al.* SOAPdenovo2: an empirically improved memory-efficient short-read de novo assembler. *GigaScience* **1**, 18, doi:10.1186/2047-217x-1-18 (2012).

2 Fu, L., Niu, B., Zhu, Z., Wu, S. & Li, W. CD-HIT: accelerated for clustering the next-generation sequencing data. *Bioinformatics (Oxford, England)* **28**, 3150-3152, doi:10.1093/bioinformatics/bts565 (2012).

3 Li, W. & Godzik, A. Cd-hit: a fast program for clustering and comparing large sets of protein or nucleotide sequences. *Bioinformatics (Oxford, England)* **22**, 1658-1659, doi:10.1093/bioinformatics/btl158 (2006).

4 Want, E. J., Wilson, I. D., Gika, H., Theodoridis, G., Plumb, R. S., Shockcor, J. *et al.* Global metabolic profiling procedures for urine using UPLC-MS. *Nature protocols* **5**, 1005-1018, doi:10.1038/nprot.2010.50 (2010).

5 Dunn, W. B., Broadhurst, D., Begley, P., Zelena, E., Francis-McIntyre, S., Anderson, N. *et al.* Procedures for large-scale metabolic profiling of serum and plasma using gas chromatography and liquid chromatography coupled to mass spectrometry. *Nature protocols* **6**, 1060-1083, doi:10.1038/nprot.2011.335 (2011).

6 Kanehisa, M. & Goto, S. KEGG: kyoto encyclopedia of genes and genomes. *Nucleic acids research* **28**, 27-30, doi:10.1093/nar/28.1.27 (2000).

7 Boulesteix, A. L. & Strimmer, K. Partial least squares: a versatile tool for the analysis of high-dimensional genomic data. *Briefings in bioinformatics* **8**, 32-44, doi:10.1093/bib/bbl016 (2007).

8 Wen, B., Mei, Z., Zeng, C. & Liu, S. metaX: a flexible and comprehensive software for processing metabolomics data. *BMC bioinformatics* **18**, 183, doi:10.1186/s12859-017-1579-y (2017).

9 Chen, J.-J., Bai, S.-J., Li, W.-W., Zhou, C.-J., Zheng, P., Fang, L. *et al.* Urinary biomarker panel for diagnosing patients with depression and anxiety disorders. *Translational psychiatry* **8**, 192, doi:10.1038/s41398-018-0245-0 (2018).

10 Chen, J.-J., Xie, J., Zeng, L., Zhou, C.-J., Zheng, P. & Xie, P. Urinary metabolite signature in bipolar disorder patients during depressive episode. *Aging (Albany NY)* **11**, 1008-1018, doi:10.18632/aging.101805 (2019).

11 Lester, G. End-product regulation of the tryptophan-nicotinic acid pathway in Neurospora crassa. *J Bacteriol* **107**, 448-455 (1971).

12 Yap, I. K. S., Angley, M., Veselkov, K. A., Holmes, E., Lindon, J. C. & Nicholson, J. K. Urinary metabolic phenotyping differentiates children with autism from their unaffected siblings and age-matched controls. *Journal of proteome research* **9**, 2996-3004, doi:10.1021/pr901188e (2010).

13 Senkowski, D., Linden, M., Zubrägel, D., Bär, T. & Gallinat, J. Evidence for disturbed cortical signal processing and altered serotonergic neurotransmission in generalized anxiety disorder. *Biol Psychiatry* **53**, 304-314 (2003).

14 Belmaker, R. H. & Agam, G. Major depressive disorder. *N Engl J Med* **358**, 55-68, doi:10.1056/NEJMra073096 (2008).

15 Blacher, E., Bashiardes, S., Shapiro, H., Rothschild, D., Mor, U., Dori-Bachash, M. *et al.* Potential roles of gut microbiome and metabolites in modulating ALS in mice. *Nature* **572**, 474-480, doi:10.1038/s41586-019-1443-5 (2019).

16 Ma, W.-N., Zhou, M.-M., Gou, X.-J., Zhao, L., Cen, F., Xu, Y. *et al.* Urinary Metabolomic Study of Chlorogenic Acid in a Rat Model of Chronic Sleep Deprivation Using Gas Chromatography-Mass Spectrometry. *Int J Genomics* **2018**, 1361402, doi:10.1155/2018/1361402 (2018).

17 Xu, J.-J., Xu, F., Shen, S.-J., Li, T., Zhang, Y.-F., Shang, M.-Y. *et al.* Holistic and dynamic metabolic alterations of traditional Chinese medicine syndrome in a toxic heat and blood stasis syndrome rat model. *RSC Advances* **7**, 56471-56483, doi:10.1039/C7RA11748E (2017).

18 Ma, Y., Bao, Y., Wang, S., Li, T., Chang, X., Yang, G. *et al.* Anti-Inflammation Effects and Potential Mechanism of Saikosaponins by Regulating Nicotinate and Nicotinamide Metabolism and Arachidonic Acid Metabolism. *Inflammation* **39**, 1453-1461, doi:10.1007/s10753-016-0377-4 (2016).

19 Morris, G., Fernandes, B. S., Puri, B. K., Walker, A. J., Carvalho, A. F. & Berk, M. Leaky brain in neurological and psychiatric disorders: Drivers and consequences. *Aust N Z J Psychiatry* **52**, 924-948, doi:10.1177/0004867418796955 (2018).

20 Tsai, S. Y., Gildengers, A. G., Hsu, J. L., Chung, K. H., Chen, P. H. & Huang, Y. J. Inflammation associated with volume reduction in the gray matter and hippocampus of older patients with bipolar disorder. *J Affect Disord* **244**, 60-66, doi:10.1016/j.jad.2018.10.093 (2019).

21 Chen, J., Yan, Y., Yuan, F., Cao, J., Li, S., Eickhoff, S. B. *et al.* Brain grey matter volume reduction and anxiety-like behavior in lipopolysaccharide-induced chronic pulmonary inflammation rats: A structural MRI study with histological validation. *Brain Behav Immun* **76**, 182-197, doi:10.1016/j.bbi.2018.11.020 (2019).

22 Zheng, P., Zeng, B., Zhou, C., Liu, M., Fang, Z., Xu, X. *et al.* Gut microbiome remodeling induces depressive-like behaviors through a pathway mediated by the host's metabolism. *Mol Psychiatry* **21**, 786-796, doi:10.1038/mp.2016.44 (2016).

23 Lv, W.-J., Wu, X.-L., Chen, W.-Q., Li, Y.-F., Zhang, G.-F., Chao, L.-M. *et al.* The Gut Microbiome Modulates the Changes in Liver Metabolism and in Inflammatory Processes in the Brain of Chronic Unpredictable Mild Stress Rats. *Oxid Med Cell Longev* **2019**, 7902874, doi:10.1155/2019/7902874 (2019).
